# A phase I dose-escalation study of pulsatile afatinib in patients with recurrent or progressive brain cancer

**DOI:** 10.1093/noajnl/vdae049

**Published:** 2024-03-30

**Authors:** Tiffany M Juarez, Jaya M Gill, Annie Heng, Jose A Carrillo, Naveed Wagle, Natsuko Nomura, Minhdan Nguyen, Judy Truong, Lucia Dobrawa, Walavan Sivakumar, Garni Barkhoudarian, Daniel F Kelly, Santosh Kesari

**Affiliations:** Pacific Neuroscience Institute and Saint John’s Cancer Institute at Providence Saint John’s Health Center, Neuro-Oncology, Santa Monica, California, USA; Pacific Neuroscience Institute and Saint John’s Cancer Institute at Providence Saint John’s Health Center, Neuro-Oncology, Santa Monica, California, USA; Pacific Neuroscience Institute and Saint John’s Cancer Institute at Providence Saint John’s Health Center, Neuro-Oncology, Santa Monica, California, USA; Pacific Neuroscience Institute and Saint John’s Cancer Institute at Providence Saint John’s Health Center, Neuro-Oncology, Santa Monica, California, USA; Pacific Neuroscience Institute and Saint John’s Cancer Institute at Providence Saint John’s Health Center, Neuro-Oncology, Santa Monica, California, USA; Pacific Neuroscience Institute and Saint John’s Cancer Institute at Providence Saint John’s Health Center, Neuro-Oncology, Santa Monica, California, USA; Pacific Neuroscience Institute and Saint John’s Cancer Institute at Providence Saint John’s Health Center, Neuro-Oncology, Santa Monica, California, USA; Pacific Neuroscience Institute and Saint John’s Cancer Institute at Providence Saint John’s Health Center, Neuro-Oncology, Santa Monica, California, USA; Pacific Neuroscience Institute and Saint John’s Cancer Institute at Providence Saint John’s Health Center, Neuro-Oncology, Santa Monica, California, USA; Pacific Neuroscience Institute and Saint John’s Cancer Institute at Providence Saint John’s Health Center, Neurosurgery, Santa Monica, California, USA; Pacific Neuroscience Institute and Saint John’s Cancer Institute at Providence Saint John’s Health Center, Neurosurgery, Santa Monica, California, USA; Pacific Neuroscience Institute and Saint John’s Cancer Institute at Providence Saint John’s Health Center, Neurosurgery, Santa Monica, California, USA; Pacific Neuroscience Institute and Saint John’s Cancer Institute at Providence Saint John’s Health Center, Neuro-Oncology, Santa Monica, California, USA

**Keywords:** Afatinib, brain cancer, ErbB, EGFR, tyrosine kinase inhibitor

## Abstract

**Background:**

Afatinib (BIBW2992; Gilotrif®) is a selective and irreversible inhibitor of the epidermal growth factor receptor (ErbB; EGFR) family. It inhibits EGFR, HER2, and HER4 phosphorylation, resulting in tumor growth inhibition and regression. This phase I dose-escalation trial of pulsatile afatinib examined the safety, drug penetration into the central nervous system, preliminary antitumor activity, and recommended phase II dose in patients with progressive or recurrent brain cancers.

**Methods:**

Afatinib was taken orally once every 4 days or once every 7 days depending on dose cohort, until disease progression or unacceptable toxicity.

**Results:**

A total of 24 patients received the investigational agent and were evaluable for safety analyses, and 21 patients were evaluable for efficacy. Dosing was administered at 80 mg every 4 days, 120 mg every 4 days, 180 mg every 4 days, or 280 mg every 7 days. A recommended phase II dose of pulsatile afatinib was established at 280 mg every 7 days as there were no dose-limiting toxicities in any of the dosing cohorts and all toxicities were deemed manageable. The most common drug-related toxicities were diarrhea, rash, nausea, vomiting, fatigue, stomatitis, pruritus, and limb edema. Out of the 21 patients evaluable for efficacy, 2 patients (9.5%) exhibited partial response based on Response Assessment in Neuro-Oncology criteria and disease stabilization was seen in 3 patients (14.3%).

**Conclusions:**

Afatinib taken orally was safe and well-tolerated up to 280 mg every 7 days in brain cancer patients.

**Key Points**

1. Oral afatinib 280 mg every 7 days can be administered safely to patients with brain cancer.
2. Afatinib concentrations in plasma generally increased with escalating pulse dosing, but afatinib in CSF was undetectable.

**Importance of Study**

Aberrant epidermal growth factor receptor (EGFR) expression and signaling contribute to the growth of malignant cells and is a therapeutic target in a variety of tumor types including brain cancers. Afatinib is a second-generation EGFR family (ErbB) irreversible tyrosine kinase inhibitor approved for use in metastatic non-small-cell lung cancer as daily dosing. A pulsatile dosing strategy could potentially increase drug exposure to brain cancers while limiting systemic toxicity from higher dosing. This clinical trial evaluated intermittent dosing of afatinib in patients with brain cancer. Results of this study demonstrated the safety and tolerability of pulsatile afatinib up to 280 mg every 7 days, but limited single-agent activity suggests additional treatment strategies are needed to improve outcomes in brain cancer.

The prognosis for primary and secondary brain cancers remains poor and treatment options are limited despite significant advances in the development of targeted therapeutics. Cellular heterogeneity, redundant or compensatory signaling pathways, and development of resistance mechanisms account for some of the roadblocks, as in other cancers, but the blood-brain barrier (BBB) presents an additional challenge for delivering therapies to tumors of the central nervous system (CNS). Inadequate drug penetration into the brain parenchyma prevents concentrations at high enough levels for antitumor activity, thus several strategies are being investigated to overcome the BBB such as using peptide-drug conjugates and nanoparticles to facilitate transcytosis, transiently opening the BBB with hyperosmolar disruption, mechanical disruption of the BBB with ultrasound, radiation, or electric fields, and chemical modifications of drugs for decreased affinity to efflux transporters.^1^

Another approach to improving drug penetrability into the CNS consists of pulsatile dosing of small molecule tyrosine kinase inhibitors (TKIs) that disrupt growth factor signaling. Aberrant signaling of the epidermal growth factor receptor (EGFR) plays a crucial role in the pathogenesis of primary brain cancers such as glioblastoma and metastatic lung cancer.^2,3^ The EGFR pathway is activated with EGFR amplification and EGFRvIII mutations in over 60% of patients with glioblastoma.^4^ In addition, there is much heterogeneity with multiple other signaling pathways that may limit response with single agents. However, impaired drug delivery to the tumor due to the BBB is still the main issue. There are several examples of studies that want to address whether pulsatile dosing can improve exposure and response.

In patients with brain tumors, daily administration of first-generation EGFR TKIs gefitinib and erlotinib did not appear to reach therapeutic levels in cerebrospinal fluid (CSF),^5–7^ prompting the exploration of alternate dosing regimens. Considering that toxicity from systemic drug accumulation often precludes delivering high enough drug dosages to penetrate the CNS,^8^ pulsatile dosing could allow for much higher peak concentrations due to the direct correlation between plasma and CSF concentrations,^9^ while also allowing a recovery period for drug clearance to mitigate systemic toxicities. Furthermore, drug retention in CSF is likely to be longer than in plasma due to negligible protein binding in the CSF compared to plasma and differences in elimination half-lives.^10,11^ In several reported cases, erlotinib concentration and exposure in the CSF were found to increase with increasing pulsatile drug doses.^9,11,12^ Interestingly, the area under the curve estimations increased 24% in the CSF versus only 2% in the plasma when erlotinib increased from 150 to 600 mg.^11^ This highlights the potential advantage of increasing drug exposure more rapidly in the CNS while limiting increased drug exposure in plasma. Significant clinical and radiographic improvements have been seen in individuals, but the small number of patients reported in these case studies limits broader interpretation of outcomes and larger studies are needed.

Afatinib is a second-generation anilinoquinazoline irreversible TKI of the EGFR family (ErbB).^13,14^ Covalent binding to the catalytic domains of EGFR (C797), human EGFR (C805), and HER4 (C803) results in decreasing auto- and transphosphorylation between ErbB dimers, and thus blocks the activity of downstream signaling pathways related to growth and apoptosis suppression.^15,16^ In addition to therapeutic implications for tumors overexpressing EGFR, afatinib showed preclinical activity against EGFRvIII and EGFR mutations such as L858R/T790M that confer resistance to first-generation reversible inhibitors gefitinib, erlotinib, and lapatinib.^17^ In 2013, afatinib (Gilotrif^®^) was approved in the United States with a recommended dosing of 40 mg daily for the first-line treatment of patients with metastatic non-small-cell lung cancer (NSCLC) whose tumors have nonresistant EGFR mutations as detected by a US FDA-approved test. Afatinib was subsequently approved for the treatment of patients with metastatic, squamous NSCLC progressing after platinum-based chemotherapy.

Afatinib concentrations in plasma with daily dosing are known to have moderate to high inter-patient variability.^18^ The current dosing schedule of afatinib may not reach sufficiently high enough intratumoral drug concentrations to maximize target inhibition in the CNS. In a phase I trial of afatinib for patients with solid tumors, the maximum concentration (C_max_) of afatinib in plasma after a single dose of 40 mg was approximately 10 nM and achieved within 2 to 5 hours after dosing.^18,19^ At the time of study design, only 1 case of daily afatinib for a lung-brain metastasis patient reported afatinib concentration in the CSF to be less than 1 nM.^20^ Furthermore, in the absence of preclinical studies of afatinib in brain cancer, the 50% inhibitory concentration (IC_50_) of afatinib for lung cancer cell lines ranged from about 1 to 140 nM depending on the EGFR status.^13,21,22^

A phase I/II trial in recurrent glioblastoma reported treatment with daily afatinib a C_max_ around 24 nM in plasma and limited single-agent activity, and the addition of afatinib to protracted temozolomide did not improve progression-free survival compared to protracted temozolomide alone.^23^ Therefore, we hypothesized that pulsatile dosing of afatinib may be tolerable, lead to increased brain penetration, and increased CNS response. We report the results of a phase I dose-escalation trial of pulsatile afatinib administered orally to adult patients with brain cancer.

## Materials and Methods

### Patient Eligibility

Eligible patients were ≥18 years old with a histologically confirmed diagnosis of any tumor or cancer in the CNS (eg, glioblastoma, anaplastic astrocytoma, anaplastic oligodendroglioma, anaplastic mixed oligoastrocytoma, low-grade gliomas, brain metastases, meningiomas, leptomeningeal metastases, chordomas, pituitary tumors, or medulloblastomas) in the dose-escalation phase, and high-grade glioma with EGFR aberrations in the expansion cohorts. Patients having progressed on prior standard therapy or, in the case of meningioma, had no other standard therapy option, as well as patients with recurrent disease, were included. Patients had Karnofsky Performance Status scores ≥ 60%, adequate bone marrow function (absolute neutrophil count ≥ 1.5 × 10^9^/L, platelet count ≥ 100 × 10^9^/L, hemoglobin ≥ 9.0 g/dL), AST/SGOT and ALT/SGPT ≤ 2.5 × institution’s upper limit of normal (ULN), total bilirubin ≤ 1.5 × ULN, and alkaline phosphatase ≤ 2.5 × ULN (unless considered tumor-related). Women of child-bearing potential and men with partners of child-bearing potential agreed to use adequate contraception while in the study.

Patients were excluded if they had not recovered from acute toxic effects of prior therapy and had received investigational agents, surgery, whole brain radiation therapy, or cytotoxic therapy within 28 days (within 42 days for nitrosourea, 23 days for temozolomide, 21 days for procarbazine, irinotecan, or topotecan, or14 days for vincristine). Patients were also excluded if they had received hormonal therapy within 14 days or non-cytotoxic agents within 7 days. Those with prior or concomitant malignancies at other sites, except effectively treated non-melanoma skin cancers, carcinoma in situ of the cervix, ductal carcinoma in situ, or effectively treated malignancy that has been in remission for more than 3 years and considered to be cured were not allowed to participate. Patients were excluded if they had known hypersensitivity to afatinib or its excipients; known preexisting interstitial lung disease; known active hepatitis B or C infection; known human immunodeficiency virus carrier; any history or presence of poorly controlled gastrointestinal disorders that could affect the absorption of study drug; a severe or uncontrolled concurrent medical disorder; impaired cardiac function; were on enzyme-inducing anti-epileptic drugs; were pregnant or nursing females; or had prior participation in a blinded afatinib clinical study.

Approval by the Western Institutional Review Board (#20161975) was obtained, and the study was conducted at Saint John’s Cancer Institute in accordance with the Declaration of Helsinki and the International Conference on Harmonization Good Clinical Practice Guidelines. All patients provided written informed consent. The clinical trial was registered on ClinicalTrials.gov (NCT02423525).

### Treatment Regimen and Dose Escalation

Afatinib was administered as a film-coated tablet that contained afatinib as dimaleate salt during each cycle of 28 days. Dose-escalation levels were 80 mg every 4 days (cohort 1), 120 mg every 4 days (cohort 2), 180 mg every 4 days (cohort 3), or 280 mg every 7 days (cohort 4). Expansion cohorts occurred for dose cohorts 3 and 4.

### Definition of Dose-Limiting Toxicity and Maximum Tolerated Dose

The opening of a new cohort followed a standard 3 + 3 dose-escalation design and was guided by assessment of all grade toxicities and trends in adverse events seen in subsequent dosing cycles. Cohort expansion to 6 patients was required if 1 dose-limiting toxicity (DLT) was reported, and dose escalation would stop if 2 DLTs were observed in those 6 patients. Safety and clinical data were reviewed through day 28 prior to the opening of each cohort level. DLT was defined as any possible drug-related, clinically relevant, grade 3 or 4 non-hematologic toxicity (except alopecia or unmedicated nausea/vomiting), grade 3 nausea, vomiting, or diarrhea of any duration lasting > 24 hours despite standard prophylaxis and/or treatment, grade 4 diarrhea and vomiting of any duration, grade 3 febrile neutropenia (defined as ANC  1000/mm^3^ with a single temperature of > 38.3° C or a sustained temperature of ≥ 38.3° C for more than 1 hour), grade 4 febrile neutropenia of any duration, grade 4 neutropenia lasting > 7 days (defined as a neutrophil count of < 500/mm^3^), grade 4 thrombocytopenia or thrombocytopenia with clinically significant bleeding, grade 4 anemia of any duration, and any clinically significant toxicity that precluded administration of the next scheduled dose beyond 14 days, or dose reduction for any reason. The maximum tolerated dose (MTD) was defined as the highest dose tested in which fewer than 33% of patients experienced a DLT. Review of study data led to expansion of dose cohort 3 while dose cohort 4 was evaluated. Dose cohort 4 was also expanded in the absence of DLTs. The recommended phase II dose was defined as a dose equal to or below the MTD and accounted for any cumulative or delayed toxicity beyond the DLT observation period.

### Safety

Safety evaluations of hematology and chemistry were conducted weekly on days 1, 8, and 15 in cycles 1 and 2 and every 2 weeks thereafter. Physical examination, vital signs collection, performance status assessment, neurological exam, and coagulation level occurred at the start of every cycle. An ECG and chest X-ray were performed at baseline prior to starting treatment. Toxicities were assessed using NCI CTCAE version 4.03.

### Pharmacokinetic Analysis

Pharmacokinetic (PK) data were obtained to guide optimal dose of afatinib. The time of maximum plasma concentration is reported between 2 and 5 hours after dosing.^18,19^ Blood samples (10 mL in ethylenediaminetetraacetic acid drawing tubes) were collected before the afatinib dose and at 3 hours (± 2 hours) after the dosing of afatinib on days 8, 15, or 22 of cycle 1, and on day 1 of cycle 2. Cerebrospinal fluid (CSF) samples (≤10 mL with 1% citric acid added to prevent adsorption loss) were collected via lumbar puncture to measure afatinib concentrations 3 hours (± 2 hours) post-drug administration once during cycle 1, and prior to drug administration once during the first 2 weeks of cycle 2.

Bioanalysis of afatinib plasma and CSF concentrations were determined using a validated method of high-pressure liquid chromatography with tandem mass spectrometry and performed at Anthem Biosciences.

### Tumor Response

Tumor response was assessed by MRI after every 2 treatment cycles, or earlier if clinically indicated, according to the Response Assessment in Neuro-Oncology criteria.

## Results

### Patients and Treatment

Between December 2016 and August 2020, 26 patients with brain cancer consented to the study; 2 were screen failures and 24 were enrolled. Descriptive analysis of baseline patient characteristics is summarized in Table 1 and provided by individual patients in Supplementary Table S1. At study entry, median age was 60 (range 33–82), 75% of patients were male, and 16 patients had glioblastoma, 3 had chordoma, 2 had brain metastases from breast cancer, and 1 each had gliosarcoma, anaplastic mixed oligoastrocytoma, and meningioma. Patients were administered afatinib in sequential dose cohorts at 80 mg every 4 days (*n* = 3), 120 mg every 4 days (*n* = 3), 180 mg every 4 days (*n* = 7), and 280 mg every 7 days (*n* = 11).

**Table 1.** Demographics and Baseline Characteristics of All Treated Patients (*N* = 24)

Characteristic	No. of patients	%
*Age, years*		
Median	60	
Range	33–82	
*Gender*		
Female	6	25.0
Male	18	75.0
*Racial origin*		
American Indian or Alaskan Native	1	4.2
Asian	1	4.2
White	21	87.5
Declined to report	1	4.2
*Pathology*		
Anaplastic mixed oligoastrocytoma	1	4.2
Brain metastases	2	8.4
Chordoma	3	12.5
Glioblastoma	16	66.7
Gliosarcoma	1	4.2
Meningioma	1	4.2
*Karnofsky performance status*		
90	12	50.0
80	3	12.5
70	9	37.7
*No. of prior regimens*		
Median	2.5	
Range	1–10	

### Safety and Tolerability

The most common adverse events possibly attributed to afatinib in patients were diarrhea (75%), nausea (37.5%), vomiting (37.5%), acneiform rash (37.5%), rash (25%), fatigue (25%), anorexia (25%), oral mucositis (20.8%), pruritus (16.7%), edema limbs (16.7%), maculopapular rash (20.8%), constipation (12.5%), fever (8.3%), and confusion (8.3%). The remaining adverse events occurred at a frequency of 1 patient each (4.2%). Grade 3 adverse events possibly attributed to afatinib included acute kidney injury (*n* = 2 in one patient), diarrhea (*n* = 1), deep vein thrombosis (*n* = 1), edema limbs (*n* = 1), gastroenteritis (*n* = 1), metabolic acidosis (*n* = 1), metabolic encephalopathy (*n* = 1), acneiform rash (*n* = 1), and maculopapular rash (*n* = 1). Grade 3 acneiform rash occurred in cohort 2, maculopapular rash occurred in cohort 3, and the remaining grade 3 events occurred in cohort 4. There were no grade 4 or 5 adverse events attributed to afatinib, and no dose-limiting toxicities occurred. Table 2 summarizes the number of patients with possible treatment-related toxicities by dose level and CTCAE grade and total number of patients per toxicity.

**Table 2.** Number of Patients with Treatment-Related AEs, by Dose Level of Afatinib

	CTCAE grade													
	80 mg (*n* = 3)			120 mg (*n* = 3)			180 mg/kg (*n* = 7)			280 mg (*n* = 11)			Total patients (*n* = 24)	
Adverse event	1	2	3	1	2	3	1	2	3	1	2	3	No.	%
*Eye disorders*														
Bilateral blepharitis							1						**1**	**4.2**
Blurred vision							1						**1**	**4.2**
*Gastrointestinal disorders*														
Constipation							1			2			**3**	**12.5**
Diarrhea	1			2	1		4	2		9	5	1	**18**	**75**
Dyspepsia										1			**1**	**4.2**
Gastroenteritis												1	**1**	**4.2**
GERD											1		**1**	**4.2**
Mucositis oral							2	1		3			**5**	**20.8**
Nausea	1			1	1		2	2		3	1		**9**	**37.5**
Vomiting					1		3	1		4	3		**9**	**37.5**
*General disorders and administration site conditions*														
Edema limbs							1			1	2	1	**4**	**16.7**
Fatigue		1		1				1			4		**6**	**25**
Fever				1						1			**2**	**8.3**
Gait disturbance											1		**1**	**4.2**
*Infections and infestations*														
Candida Intertrigo							1						**1**	**4.2**
Thrush (oral)							1						**1**	**4.2**
*Investigations*														
Thrombocytopenia							1	1					**1**	**4.2**
*Metabolism and nutrition disorders*														
Anorexia				1	2		2	3					**6**	**25**
Hypokalemia										1			**1**	**4.2**
Metabolic acidosis												1	**1**	**4.2**
Metabolic encephalopathy												1	**1**	**4.2**
*Musculoskeletal and connective tissue disorders*														
Bilateral intermittent leg cramps										1			**1**	**4.2**
*Nervous system disorders*														
Amnesia								1			1		**1**	**4.2**
Dysgeusia													**1**	**4.2**
Memory impairment					1								**1**	**4.2**
Headache										1			**1**	**4.2**
Paresthesia (lower extremities)				1									**1**	**4.2**
*Psychiatric disorders*														
Confusion							1				1		**2**	**8.3**
Insomnia					1								**1**	**4.2**
*Renal and urinary disorders*														
Acute kidney injury												1	**1**	**4.2**
*Respiratory thoracic and mediastinal disorders*														
Dyspnea										1			**1**	**4.2**
*Skin and subcutaneous tissue disorders*														
Ingrown nail										1			**1**	**4.2**
Palmar-Plantar Erythrodysesthesia						1							**1**	**4.2**
Pruritus				1	1			1		1			**4**	**16.7**
Rash	1						1	1		1	2		**6**	**25**
Rash acneiform	1			2	1	1		1		3	1		**9**	**37.5**
Rash maculo-papular							2	1	1	3			**6**	**20.8**
Skin atrophy								1					**1**	**4.2**
Skin ulceration								1					**1**	**4.2**
*Vascular disorders*														
DVT												1	**1**	**4.2**

Abbreviations: AE, adverse event; CTCAE, Common Terminology Criteria for Adverse Events; DVT, deep vein thrombosis; GERD, gastroesophageal reflux disease.

Serious adverse events considered at least possibly related to afatinib included a grade 1 fever, grade 3 gastroenteritis, and grade 3 diarrhea. A 56-year-old male with glioblastoma in cohort 4 initiated afatinib 280 mg every 7 days and within 2.5 months experienced grade 1 fever classified as an SAE. He was hospitalized for 3 days during which no infection was identified, and fever resolved on day 3. A 60-year-old male with glioblastoma in cohort 4 initiated afatinib 280 mg every 7 days and within 2 weeks experienced grade 3 gastroenteritis. His symptoms resolved within 3 days and the afatinib was reduced from 280 mg every 7 days to 200 mg every 7 days. The same patient later experienced grade 3 diarrhea 1 month after starting treatment, was hospitalized for 4 days before the diarrhea resolved and discontinued study treatment. The recommended phase II dose was identified as 280 mg every 7 days.

### Pharmacokinetics of Afatinib

**Figure 1.**
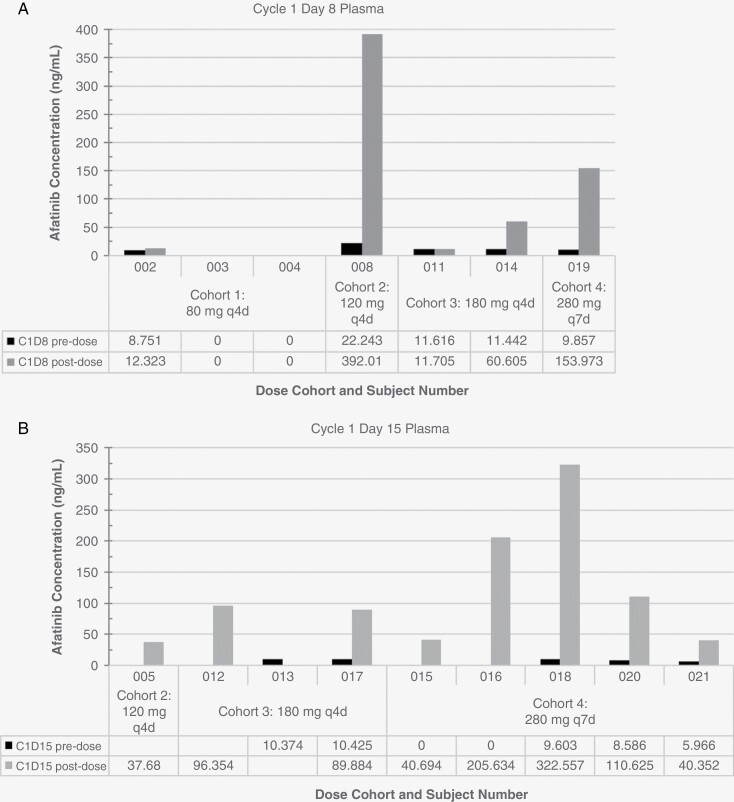
Afatinib concentrations in plasma. Afatinib levels were measured in plasma collected prior to dosing and approximately 3 hours after dosing on cycle 1 day 8 (A) and cycle 1 day 15 (B).

Following oral administration of afatinib, the geometric mean maximum concentrations of afatinib (C_max_) of 0 to 392 ng/mL in plasma were reached at 3 hours (± 2 hours) post-end of dosing (Table 3). Plasma samples for subjects 002–006, 008, and 010–021 were sent for analysis. Longitudinal data sets were incomplete. Plasma concentrations on cycle 1 day 8 and cycle 1 day 15 are visualized in Figure 1. Based on limited data, there appeared to be a higher Cmax in plasma with pulse dosing. CSF specimens collected from 8 patients approximately 3 hours after dosing in the first cycle were sent for analysis; observed concentrations of afatinib in CSF samples were below the lower limit of quantification in CSF (1.015 ng/mL; data not shown).

**Table 3.** Afatinib Concentration (ng/mL) in Blood at Indicated Time Points

Cohort	Subject ID	Dosing	C1D8	C1D15	C1D22	C2D1	C3D1	C4D1	EOT
Cohort 1: 80 mg q4d	002	pre	8.751	—	—	11.693	—	—	<LLOQ (EoC2)
		post	12.323	—	—	—	—	—	—
	003	pre	<LLOQ	—	17.318	—	—	—	—
		post	<LLOQ	—	30.096	—	—	—	—
	004	pre	<LLOQ	—	—	<LLOQ	—	—	—
		post	<LLOQ	—	—	—	—	—	—
Cohort 2: 120 mg q4d	005	pre	—	—	—	7.18	—	—	—
		post	—	37.68	—	—	—	—	—
	006	pre	—	<LLOQ	—	10.104	<LLOQ	11.223	—
		post	—	66.156	—	—	—	—	—
	008	pre	22.243	—	—	—	—	—	—
		post	392.01	—	—	—	—	—	—
Cohort 3: 180 mg q4d	010	pre	—	—	43.226	—	—	—	—
		post	—	—	324.265	—	—	—	—
	011	pre	11.616	—	—	—	19.265	—	—
		post	11.705	—	—	—	—	—	—
	012	pre	—	<LLOQ	—	<LLOQ	—	—	6.402 (EoC4)
		post	—	96.354	—	85.834	—	—	—
	013	pre	—	10.374	—	41.964	—	6.787	—
		post	—	—	—	—	—	—	—
Expansion Cohort 3: 180 mg q4d	014	pre	11.442	—	—	—	—	—	—
		post	60.605	—	—	—	—	—	—
	017	pre	—	10.425	-	5.708	<LLOQ	6.264	-
		post	—	89.884	—	—	—	—	—
Cohort 4: 280 mg q7d	015	pre	—	<LLOQ	—	—	—	—	<LLOQ (EoC1)
		post	—	40.694	—	—	—	—	—
	016	pre	—	<LLOQ	—	—	—	—	—
		post	—	205.634	—	—	—	—	—
	018	pre	—	9.603	—	22.084	14.169	11.731	10.379 (EoC4)
		post	—	322.557	—	340.709	—	—	—
Expansion Cohort 4: 280 mg q7d	019	pre	9.857	—	—	11.805	—	—	<LLOQ (EoC2)
		post	153.973	—	—	—	—	—	—
	020	pre	—	8.586	—	11.278	—	—	—
		post	—	110.625	—	—	—	—	—
	021	pre	—	5.966	—	<LLOQ	—	—	<LLOQ (C2D15)
		post	—	40.352	—	—	—	—	—

Abbreviations: EOT, end of treatment; EoC, end of cycle; q, every; d, days; LLOQ, lower limit of quantitation.

### Antitumor Activity

Out of 24 patients, 3 patients withdrew from the study prior to a response assessment for reasons other than disease progression and 21 patients were evaluated for response. Transient partial response was observed in (9.5%) 2 patients (Supplementary Figure S1). Patient 006 in cohort 2 had *EGFR*-amplified glioblastoma having progressed on 2 prior therapies of standard chemoradiation followed by 11 cycles of adjuvant temozolomide and 1 cycle of an investigational proteasome inhibitor on a clinical trial. Patient 018 in cohort 4 had multiple sub-cm brain metastases from ERBB2-amplified and ERBB3-mutated breast cancer and was treated on study after receiving docetaxel, trastuzumab, and pertuzumab for primary and metastatic disease along with cerebellar radiation.

**Figure 2.**
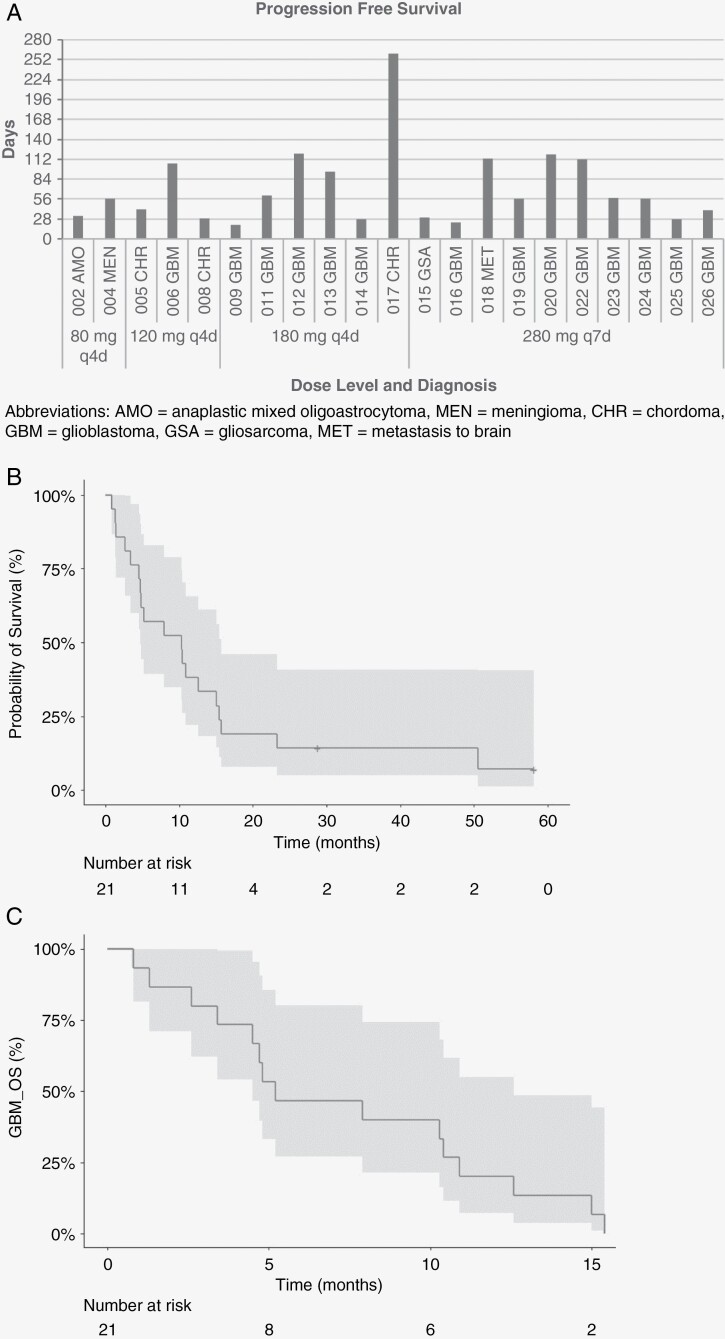
Kaplan–Meier curves of survival probability among patients treated with afatinib. (A) Progression-free survival for efficacy-evaluable patients by afatinib dose level and diagnosis. (B) Survival probability among efficacy-evaluable patients treated with pulsatile afatinib, with confidence interval. (C) Survival probability of patients with glioblastoma, with confidence interval. Abbreviations: AMO, anaplastic mixed oligoastrocytoma; MEN, meningioma; CHR, chordoma; GBM, glioblastoma; GSA, gliosarcoma; MET, brain metastasis

Disease stabilization was seen in 3 patients (14.3%), 2 of whom had glioblastoma (1 EGFR amplification, 1 EGFR wild type) and 1 who had EGFR wild-type chordoma (Table 4). Sixteen patients exhibited progressive disease, 5 of whom had EGFR amplification, 3 with EGFR amplification and EGFRvIII mutation, 3 with EGFR mutations (1 vIII, 1 G1783T, 1 missense), 3 with EGFR wild type, and 2 undetermined. Overall, median progression-free survival was 2.0 months (Figure 2A). Median overall survival was 10.3 months (95% CI: 4.7–15.7) for all patients (Figure 2B) and 5.2 months (95% CI: 4.5–12.6) for glioblastoma. (Figure 2C).

**Table 4.** Number of Cycles, PFS, and Best Response by Dose Level (*N* = 24)

Dose cohort	No. cycles		PFS (days)		Best Response				No. response-evaluable
	Median	Range	Median	Range	CR	PR	SD	PD	
1 (80 mg every 4 days)	1.3	1–2	44	32–56	0	0	0	2	2
2 (120 mg every 4 days)	1.5	1–4	42	29–106	0	1	0	2	3
3 (180 mg every 4 days)	2.2	1–9.3	78	20–261	0	0	2	4	6
4 (280 mg every 7 days)	2.0	1–4.3	56	23–119	0	1	1	8	10

Abbreviations: PFS, progression-free survival; CR, complete response; PR, partial response; SD, stable disease; PD, progressive disease.

## Discussion

Targeting aberrant growth factor signaling with small molecule inhibitors is a validated approach for many types of cancer but continuous low-dosing administration may not be adequate for reaching therapeutic concentrations for CNS cancers. We conducted a phase I dose-escalation trial of afatinib administered every 4 or 7 days in attempt to increase drug penetration and antitumor activity.

Pulsatile dosing of afatinib was generally well-tolerated. Consistent with known side effects of afatinib, the most common adverse events with pulsatile dosing were diarrhea, rash, nausea, vomiting, stomatitis, and pruritus as well as fatigue, anorexia, and limb edema. No grade ≥4 or dose-limiting toxicities were observed.

Based on the incomplete pharmacokinetic dataset, pulse dosing appeared to increase peak afatinib concentration in the blood while pre-dose (trough) levels remained consistently under 50 nM. High variability was observed between patients and between dose levels, with some patients having unmeasurable levels of afatinib to one patient demonstrating approximately 0.8 μM in the 120 mg every 4 days cohort. Unfortunately, afatinib was not detected in CSF samples and it is unclear whether levels were simply below the lower limit of quantification (1.015 ng/mL; 2.09 nM) or whether other factors contributed such as permeability of the BBB based on the disease type or the sampling time. In an 11-patient study of afatinib 40 mg daily for leptomeningeal metastases from NSCLC, the median concentration of afatinib in CSF collected at steady-state on day 8 was 2.9 nM (range, not evaluable—6.0 nM).^24^ Two other cases of leptomeningeal carcinomatosis treated with daily afatinib reported afatinib concentrations in the CSF of 0.05 ^25^ and 1 nM.^20^ A preclinical study published after the start of the trial reported the inhibitory concentration of 25% (IC_25_) values were approximately 2 and 1 μM of afatinib for U87MG and U87EGFRvIII human glioblastoma cells, respectively.^26^ From the available measurements in our study, afatinib levels did not reach micromolar levels in plasma, and were therefore unlikely to reach micromolar levels in the CNS. Furthermore, in an orthotopic mouse model of NSCLC treated with 30 mg/kg afatinib, CSF collected from the dura through the atlanto-occipital membrane demonstrated peak concentrations one hour after dosing and a concentration ratio of CSF to plasma of 4.2% that decreased over time.^27^ Incorporating more rigorous assessment of CNS drug levels into clinical trials is needed to provide better understanding of CNS pharmacokinetics.

The dose-escalation part of the study permitted enrollment of unselected patients with brain cancer and genetic testing was not mandatory at the time the trial was initiated. However, the expansion cohorts for afatinib 180 mg every 4 days and 280 mg every 7 days required enrollment of high-grade glioma with *EGFR* aberrations. Overall, 2 patients experienced partial response, 1 with *EGFR*-amplified glioblastoma and 1 with multiple brain metastases from ERBB2-amplified and ERBB3-mutated breast cancer. Subgroup assessment of patients with glioblastoma (*n* = 16) showed a progression-free survival rate at 6 months (PFS6) was 0%. *EGFR* genetic testing in the expansion cohorts revealed glioblastomas with EGFR amplification, *EGFR*vIII mutations, and *EGFR* mutations not vIII (V774M, G1793T). The small sample size, therefore, limited inferences about non-glioblastoma tumor types and more in-depth molecular profiling should be included in future studies to provide additional insights.

In summary, this dose-finding study of pulsatile afatinib for adults with brain cancer established the safety and tolerability of afatinib in this patient population and identified a recommended phase II dose of 280 mg every 7 days. Limited activity was seen in this heterogeneous population, and additional strategies will be needed in future studies of pulsatile afatinib for the treatment of brain tumors. Future considerations include selecting patients with activated EGFR pathway by protein expression, administration of afatinib prior to tumor resection for a direct measurement of afatinib in the tumor since CSF levels were unmeasurable, and investigations with pulsatile dosing of later-generation EGFR TKIs, such as osimertinib, which was not available at the start of this study. As there is known variability in afatinib concentrations between patients and intratumoral levels may be different than CSF levels, further resolution of CNS pharmacokinetics is needed.^28^ Additionally the infiltrative nature and tumor heterogeneity of CNS tumors such as glioblastoma will likely require a combination approach to truly achieve high and durable activity.

## Supplementary Material

vdae049_suppl_Supplementary_Appendix

## Data Availability

The datasets analyzed during the current study are available from the corresponding author on reasonable request.
