# Supplementary material for: A phase I dose-escalation study of pulsatile afatinib in patients with recurrent or progressive brain cancer
Source: Neurooncol Adv. 2024 Mar 30;6(1):vdae049. doi: 10.1093/noajnl/vdae049 (PMC11046985; doi:10.1093/noajnl/vdae049)
Supplement: vdae049_suppl_Supplementary_Appendix [file vdae049_suppl_supplementary_appendix.docx]

**Supplementary Appendix**

**A Phase I Dose Escalation Study of Pulsatile Afatinib in Patients with Recurrent or Progressive Brain Cancer**

Tiffany M. Juarez, Jaya M. Gill, Annie Heng, Jose A. Carrillo, Naveed Wagle, Natsuko Nomura, Minhdan Nguyen, Judy Truong, Lucia Dobrawa, Walavan Sivakumar, Garni Barkhoudarian, Daniel F. Kelly, Santosh Kesari

Pacific Neuroscience Institute and Saint John’s Cancer Institute at Providence Saint John’s Health Center, Santa Monica, CA, USA

**SUPPLEMENTARY APPENDIX**

**Table of Contents**

[Table S1. Patient demographics, diagnosis, and EGFR status at treatment initiation. 3](#_Toc147324706)

[Figure S1. Magnetic resonance imaging of patients 006 and 018. 4](#_Toc147324707)

# Table S1. Patient demographics, diagnosis, and EGFR status at treatment initiation.

| **Patient ID#** | **Age** | **Gender** | **Race** | **Ethnicity** | **Brain Cancer Diagnosis** | **No. prior regimens** | **Baseline KPS** | **EGFR status** |
| --- | --- | --- | --- | --- | --- | --- | --- | --- |
| 002 | 82 | Female | White | Not Hispanic or Latino | Anaplastic mixed oligoastrocytoma | 3 | 80 | not available |
| 003 | 77 | Male | White | Not Hispanic or Latino | Glioblastoma | 2 | 90 | Wildtype |
| 004 | 42 | Male | American Indian or Alaska Native | Hispanic or Latino | Meningioma | 1 | 90 | Wildtype |
| 005 | 55 | Male | White | Not Hispanic or Latino | Chordoma | 3 | 90 | Wildtype |
| 006 | 64 | Male | White | Not Hispanic or Latino | Glioblastoma | 2 | 90 | EGFR amplification |
| 008 | 61 | Male | White | Not Hispanic or Latino | Chordoma | 10 | 70 | Wildtype |
| 009 | 38 | Male | White | Not Hispanic or Latino | Glioblastoma | 2 | 70 | not available |
| 010 | 61 | Female | White | Not Hispanic or Latino | Brain metastases | 4 | 80 | not available |
| 011 | 66 | Male | White | Not Hispanic or Latino | Glioblastoma | 3 | 90 | EGFRvIII |
| 012 | 54 | Male | Declined to report | Not Hispanic or Latino | Glioblastoma | 2 | 90 | EGFR amplification |
| 013 | 57 | Male | White | Not Hispanic or Latino | Glioblastoma | 6 | 70 | EGFR amplification |
| 014 | 40 | Male | White | Not Hispanic or Latino | Glioblastoma | 4 | 70 | EGFR amplification |
| 015 | 33 | Female | White | Not Hispanic or Latino | Gliosarcoma | 5 | 90 | EGFR missense mut |
| 016 | 44 | Male | Asian | Not Hispanic or Latino | Glioblastoma | 4 | 80 | EGFR amplification, EGFRvIII |
| 017 | 60 | Male | White | Not Hispanic or Latino | Chordoma | 1 | 90 | Wildtype |
| 018 | 54 | Female | White | Not Hispanic or Latino | Brain metastases, > 30 sub-cm lesions | 1 | 90 | Wildtype |
| 019 | 56 | Male | White | Hispanic or Latino | Glioblastoma | 2 | 70 | EGFR amplification, EGFRvIII |
| 020 | 68 | Male | White | Not Hispanic or Latino | Glioblastoma | 1 | 70 | Wildtype |
| 021 | 60 | Male | White | Not Hispanic or Latino | Glioblastoma | 3 | 70 | EGFR amplification, EGFR V7774M |
| 022 | 61 | Female | White | Not Hispanic or Latino | Glioblastoma | 1 | 90 | EGFR amplification |
| 023 | 71 | Male | White | Not Hispanic or Latino | Glioblastoma | 2 | 90 | EGFR amplification |
| 024 | 57 | Female | White | Not Hispanic or Latino | Glioblastoma | 3 | 90 | EGFR amplification |
| 025 | 70 | Male | White | Not Hispanic or Latino | Glioblastoma | 1 | 70 | EGFR amplification, EGFRvIII |
| 026 | 63 | Male | White | Not Hispanic or Latino | Glioblastoma | 3 | 70 | EGFR G1793T |


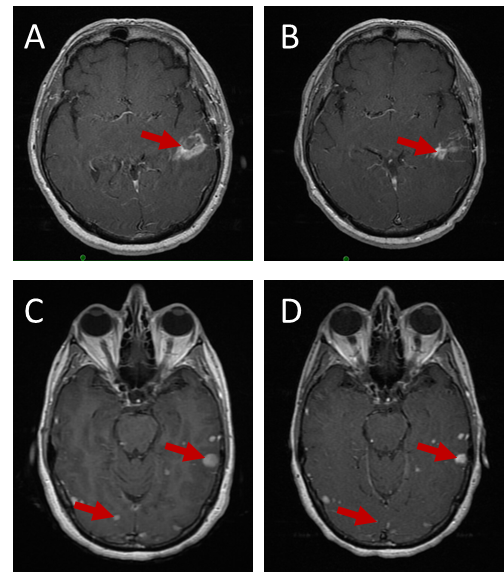


**Figure S1.** Magnetic resonance imaging of patients 006 and 018.

Brain MRI of patient 006 shows axial T1 post-gadolinium images of a left temporal glioblastoma (arrows) before treatment (A) and one month after treatment initiation with afatinib (B). Brain MRI of patient 018 shows axial T1 post-gadolinium images of multiple lesions (arrows) before treatment with afatinib (C) and two months after treatment initiation with afatinib (D).
